# The role of protein prenylation inhibition through targeting FPPS by zoledronic acid in the prevention of renal fibrosis in rats

**DOI:** 10.1038/s41598-024-68303-z

**Published:** 2024-08-07

**Authors:** Reham Hussein Mohamed, Dina S. Abdelrahim, Nesma Hussein Abdel Hay, Nesma Mohamed Fawzy, Doaa Karem M. M., Dalia Ahmed Yousef Yehia, Omnia M. AbdelMaksoud, Yomna M. Tamim

**Affiliations:** 1 Department of Clinical Pharmacology, Faculty of Medicine, Ain Shams University, Abbasia, Cairo, Egypt; 2 Department of Medical Biochemistry and Molecular Biology, Faculty of Medicine, Ain Shams University, Cairo, Egypt; 3 Department of Histology, Faculty of Medicine, Ain Shams University, Cairo, Egypt; 4 Department of Medical Physiology, Faculty of Medicine, Cairo University, Cairo, Egypt; 5 Department of Pharmacology, Faculty of Medicine, Modern Technology & Information University, Cairo, Egypt

**Keywords:** Zoledronic acid, Renal fibrosis, Protein prenylation, FPPS, NF-κB, Molecular biology, Nephrology

## Abstract

Renal fibrosis (RF) represents the most widespread pathological condition in chronic kidney disease (CKD). Recently, protein prenylation has been implicated in the fibrosis’s progression. The research examined the renoprotective effect of zoledronic acid (ZA) (50 µg/kg/week) in a rat model of carbon tetrachloride (CCl_4_)-induced RF through targeting protein prenylation. Forty Wistar male rats were split up into the control group, vehicle-treated group, model-RF group, and RF-ZA group. Mean arterial blood pressure (MBP), BUN, serum creatinine, and urine albumin–creatinine ratio (uACR), protein levels of farnesyl pyrophosphate (FPP), tumour necrosis factor-alpha (TNF-α), transforming growth factor-β (TGF-β), and malondialdehyde (MDA), and catalase and gene expression of farnesyl pyrophosphate synthase (FPPS) and nuclear factor-kB (NF-κB) were measured. Immunohistochemical staining for renal interleukin-6 (IL-6), α-smooth muscle actin (α-SMA), and caspase-3, as well as histopathological alterations, were assessed. ZA considerably ceased the reduction in MBP, markedly reduced uACR, serum creatinine, BUN, and expression of FPPS, FPP, NF-κB, TGF-β, TNF-α, and MDA, and significantly increased catalase levels compared to the model-RF rats. ZA ameliorated the CCl_4_-induced histopathological alterations and suppressed the expression of caspase-3, α-SMA, and IL-6. In conclusion, ZA preserved renal function and prevented renal fibrosis in a rat model. These were achieved through targeting protein prenylation mainly by inhibiting FPPS.

## Introduction

Renal fibrosis (RF) is frequently a consequence of progressive chronic kidney disease (CKD) and is viewed as an advanced phase of renal damage. Antifibrotics may pose challenges in treatment leading to treatment failure^1^. Additionally, over the past thirty years, the worldwide mortality rate from CKD has increased by 41.5%^2^. Thus, it is best suitable to test individuals at high risk, such as those with hypertension, diabetes, and the elderly, for early prevention of renal failure^3^.

Recent studies have connected protein prenylation with the advancement of fibrosis^4^. Protein prenylation is a crucial post-translational modification of proteins that involves protein farnesylation and geranylgeranylation. Protein prenylation is facilitated by geranylgeranyl pyrophosphate (GGPP), farnesyl pyrophosphate (FPP), and short-chain isoprenoids.

Farnesyl pyrophosphate synthase (FPPS) is a prime target for inhibiting prenylation^5^. This enzyme facilitates the joining of two molecules of isopentenyl diphosphate with dimethylallyl diphosphate in a head-to-tail manner to produce farnesyl pyrophosphate (FPP)^6^. FPPS expression is increased by oxidative stress^7^. Among the proteins that become prenylated are small GTPases, such as RhoGTPases, which require prenylation for activity^8^. RhoGTPases activate NFκB signaling. NFκB is a group of transcription factors that are always present and can be activated, which are crucial in the development of inflammation and fibrosis^9^. NF-κB directly targets inflammation by increasing the production of inflammatory cytokines such interleukin-1 (IL-1), tumor necrosis factor-alpha (TNF-α), and IL-6. It also controls cell proliferation, transformation, and apoptosis. NF-κB activation contributes to profibrogenic signaling pathways, resulting in increased transforming growth factor-β (TGF-β) signaling^10^. The NFκB pathway plays a crucial role in human disorders such as CKD and could be a promising target for therapy^11,12^.

Zoledronic acid (ZA), a strong FPPS inhibitor, is commonly used for treating osteoporosis and metastatic bone disease and has potential use in cancer treatment. ZA decreases the levels of the isoprenoid substrates FPP and GGPP that are necessary regarding the post-translational alteration of RhoGTPases and the activation of the NFκB pathway. ZA has been found to have positive effects on pulmonary and liver fibrosis by targeting FPPS, which is different from existing therapy approaches^13,14^. Thus, ZA may have a beneficial therapeutic impact on chronic kidney disease (CKD) and renal failure (RF). Nevertheless, there have been reports indicating that the usage of ZA raises significant concerns regarding its nephrotoxic effects. Some research suggested that it could harm kidney function and cause tubular toxicity more often, while other investigations found it to be harmless for the kidneys^15,16^. The purpose of this study was to evaluate the effectiveness of ZA at a dosage of 50 µg/kg/week on a rat model of carbon tetrachloride (CCl_4_)-induced RF by evaluating mean arterial blood pressure (MBP), kidney index, blood urea nitrogen (BUN) and serum creatinine levels, urine albumin–creatinine ratio (uACR), and the expression of FPPS, FPP, NF-κB, TNF-α, TGF-β, malondialdehyde (MDA), catalase in the kidneys, histopathological alterations, and immunohistochemical examination for kidney IL-6, smooth muscle actin, and caspase-3.

## Methods

### Animals and drugs

Wistar male rats, weighing between 150 and 200 g and aged between 14 and 16 weeks, were housed in a climate-controlled chamber with a 22 °C temperature and a 12 h light–dark cycle. A 1 week acclimatization interval was provided before starting the experimental regimen.

### Ethical approval

All animal procedures were carried out in accordance with ARRIVE guidelines^17^. The study was approved by the Ain Shams University, Faculty of Medicine’s Institutional Animal Ethics Committee. The application approval number was FMASU MD R115/2023, which runs according to the guidelines of the International Council on Harmonization (ICH) for Medical Science (IOMS), the United States Office for Human Research. The animal experiment was done according to the guidelines of ethical care and standard regulations.

Zoledronic acid (ZA) was supplied by Sigma Aldrich Chemicals, as a white powder as a white powder dissolved in 0.9% saline. ZA was created by combining 0.05 mL of the chemical with 0.95 mL of normal saline, resulting in a concentration of 0.05 mg/ml ZA. A transparent mixture of carbon tetrachloride (CCl_4_) and sterilized olive oil was provided in a 1:1 ratio, at a dosage of 4 mL per kilogram of total volume.

The dose of ZA to osteoporotic patients is ranging from 4 to 5 mg^18^. We extrapolated the clinical dose of the drug received by patients for osteoporosis treatment (4 mg) according to rats’ weight, metabolic rates, and treatment period protocols. For the Wistar rats, the calculated dosage was 0.60 mg/kg. Then, it was split to be administered for 12 weeks to 50 µg/kg/week^19,20^.

### Experimental design

**Figure 1:**
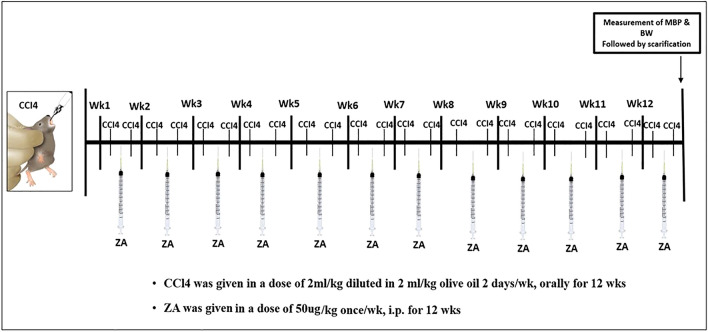
Diagrammatic illustration of the study timeline for drug administration and timing of outcome measures. *Wk* week, *MBP* mean blood pressure, *ZA* zoledronate, *CCl*_*4*_ carbon tetrachloride, *i.p.* intraperitoneal injection.

Four groups of animals were randomly divided, each containing ten rats as follows: group 1 (control): rats were given oral saline throughout the experiment; group 2 (vehicle-treated): For a period of 12 weeks, rats were given 2 mL of olive oil per kilogram of body weight orally on Monday and Thursday ; group 3 (model-RF): For a period of 12 weeks, oral ingestion of 2 mL of carbon tetrachloride (CCl_4_) and 2 mL of olive oil per kilogram of body weight on Monday and Thursday caused renal fibrosis (RF)^21,22^ and group 4 (RF-ZA): Rats received ZA intraperitoneally (i.p.) at a dose of 50 µg/kg/week in addition to the previous method of CCl_4_ at the start and during the course of the study, Fig. 1.

### Measurement of kidney index

All the experiment rats’ body weights were measured for every group. Every rat that was sacrificed had its two kidneys removed and weighed. And after that, the kidney index was determined by dividing the total weight of both kidneys to the body weight of each rat.

### Assessment of renal functions

#### Measurement of mean blood pressure (MBP)

At the end of the experiment and before rats’ scarification, (45 mg/kg, i.p.) sodium pentobarbital was used for the anesthesia of rats, followed by insertion of the tube intratracheally and opening of the wall of the anterior abdomen. By cannulating the abdominal aorta with a polyethylene catheter (PE50) loaded with heparinized saline (100 U/ml) and attached to a physiological pressure transducer (SP844) manufactured by Power Lab (AD Instruments, Germany), the mean blood pressure (MBP) was measured.

#### Measurement of urine albumin–creatinine ratio (uACR)

The collection of 24 h rats’ urine was done in metabolic cages before scarification. The concentration of urinary microalbumin (mg/24 h) was measured using a rat albumin ELISA kit (Gen Way Biotech, San Diego). The concentration of urinary creatinine (g/24 h) was measured using QuantiChrom Creatinine ELISA kit (BioAssay Systems, USA) and Creatinine Jaffè ELISA kit (Spectrum, Egyptian Company for Biotechnology). The procedures were performed in compliance with the manufacturer’s guidelines.

#### Measurement of serum creatinine and blood urea nitrogen (BUN) levels

Using aseptic techniques, blood was drawn from all experimentally sedated animals through retroorbital perforation. The specimens were centrifuged at 1000*g* for 15 min after being left to coagulate for 2 h at room temperature. After extraction, the serum specimens were kept at − 80 °C. The concentration of creatinine in serum (nmol/ml) was calculated with a creatinine (Rat) ELISA kit (BioVision, USA, Cat. No. E4370-100). The concentration of urea in serum (nmol/ml) was measured using rat blood urea nitrogen (BUN) ELISA kit (SunLong Biotech Co., Cat. No. SL1053Ra). The procedures were performed per the guidelines provided by the manufacturer.

### Molecular studies

Left kidneys were removed after scarification, and renal tissue homogenization was done using phosphate buffer saline (10% PBS). To 4 g of tissue, 16 mL of 10% PBS were added and spent 20 min centrifuging at 2000*g*, followed by freezing of the supernatant at − 80 °C till examination. Assessment of the specimens was done for the subsequent criteria.

#### Measurement of Malondialdehyde (MDA) and catalase levels in kidney tissue

Quantitative measurement of MDA was performed using lipid peroxidation (MDA) colorimetric/fluorometric assay kit (BioVision, Cat. No. K739-100, USA), and estimation of catalase activity was done by using catalase activity colorimetric/fluorometric assay kit (BioVision, Cat. No. K773-100, USA).

#### Measurement of tumor necrosis factor-alpha (TNF-α) and transforming growth factor-β (TGF-β) in kidney tissue

The tissue level of TNF-α was assessed by the ELISA MAX^™^ Deluxe Set (Biolegend et al., Cat. No. 438206). In contrast, tissue TGF-β was quantified using an ELISA kit (Cloud-Clone Corp., USA, Cat. No. SEA124Ra) in compliance with the manufacturer’s guidelines.

#### Measurement of farnesyl pyrophosphate (FPP) in kidney tissue

The concentration of FPP was estimated in kidney tissue with the use of rat squalene synthase, FDFT1 ELISA kit (Cat. No: MBS9331891).

#### Gene expression analysis for farnesyl pyrophosphate synthase (FPPS) and nuclear factor-κB (NF-κB) by real-time quantitative polymerase chain reaction (RT-qPCR)

##### RNA extraction

The whole RNA has been extracted from homogenized tissues of each group using Direct-zol RNA Miniprep Plus (Cat. No: R2072, ZYMO RESEARCH CORP., the United States), and then both quality and quantity were assessed using a Beckman dual spectrophotometer (USA).

##### Real-time PCR

Using a SuperScript IV One-Step RT-PCR kit (Cat# 12594100, Thermo Fisher Scientific, Waltham, MA, USA), extracted RNA was reverse transcribed to cDNA and then subjected to PCR. Primer’s sequence used for amplification of NFκB gene was forward 5′-GTCTCAAACCAAACAGCCTCAC -3′, and reverse 5′- CAGTGTCTTCCTCGACATGGAT -3′ (gene bank accession number NM_199267.2). FPPs gene was forward 5′-TGGTGTGTAGAACTGCTCCAGGCTTTCTTC-3′, and reverse 5′- ACACTGGGGTCTCCAAAGAGATCAAGGTAG-3′ (gene bank accession number NM_031840), and GAPDH housekeeping gene was forward 5′- CCTCGTCTCATAGACAAGATGGT -3′ and reverse 5′- GGGTAGAGTCATACTGGAACATG -3′ (gene bank accession number NM_001394060.2). On a plate with 96 wells, the StepOne instrument (Applied Biosystems, USA) was used in the following way for a thermal profile: reverse transcription is carried out for 10 min at 45 ºC, RT inactivation for 2 min at 98 ºC, and initial denaturation is carried out in 40 cycles of 10 s at 98 ºC, 10 s at 55 ºC, and 30 s at 72 ºC for the amplification step. The data for the target genes and housekeeping genes was provided in cycle threshold (Ct) following the RT-PCR run. Normalization of target gene expression variation; NFκB and FPPS were carried out relating for the expression for the mean critical threshold (CT) values of the GAPDH housekeeping gene by the ΔΔCt method. The 2^−∆∆Ct^ method is utilized to calculate the relative quantitation (RQ) of every target gene^23^. Figures S1 and S2.

### Histopathological and immunohistochemical study

The right kidney was taken from all animals through an abdominal incision. Kidneys were divided longitudinally into two halves. Then, they were fixed in 10% formalin over 5 days. Following a dehydration process using increasing alcohol grades, the samples were cleaned in xylene and placed in paraffin. Sections of paraffin were cut in serial at 5 μm thickness^24^. Several procedures were applied to paraffin sections: Haematoxylin and eosin (H&E) and picro sirius red stain (ab150681; Abcam, MA, USA) for demonstration of collagen fibers. Additional sections were sliced and put on positively charged slides for immune-histochemical staining with an anti-caspase-3 antibody using the avidin–biotin peroxidase method [Mouse monoclonal—ABM1C12] (ab208161; Abcam, MA, USA), Anti-IL-6 antibody [mouse monoclonal 1.2-2B11-2G10] (ab9324; Abcam, MA, USA) and anti-alpha smooth muscle actin (α SMA) antibody [mouse monoclonal [1A4] (ab7817; Abcam, MA, USA). We used hematoxylin to counterstain the slides.

Similar procedure was followed when processing negative controls, but without using the primary antibody. Positive control sections for caspase-3, IL-6 and α-SMA were examined utilizing sections of the tonsil, kidney, and colon (respectively) and this was done according to the same protocol.

Caspase-3 reaction was done for demonstration of apoptosis. IL-6 was done for demonstration of inflammatory cells and α-SMA was done for demonstration of smooth muscle cells in vascular structures as well as myofibroblasts. The positive reaction appeared as brown cytoplasmic reaction.

### Histo-morphometric studies and analytical statistics

A computer with the Leica Q win V.3 image analyzer program installed was used. The Leica DM2500 microscope (Wetzlar, Germany) was linked to the computer. Every group of animals underwent morphological analysis. Five distinct slides were obtained from each animal, and measurements were made from them. For every slide, five randomly chosen, not-overlapping fields were looked at. The following parameters were measured:

- 1-The diameter of the PCT in H & E-stained sections (X400).
- 2-Area % of collagen fibers in picrosirius red sections with stains (X200).
- 3-Area % of caspase-3 immune-histochemical reaction (X400).
- 4-Area % of IL-6 immune-histochemical reaction (X400).
- 5-Area % of α-SMA in immune-histochemical stained sections (X400).

Every study’s morphometric data was gathered and then statistically examined. The SPSS statistical software, version 21, was used to determine the mean value and the standard deviation (SD) for each group (IBM Inc., Chicago, Illinois, USA). One-way analysis of variance (ANOVA) was used to statistically analyze the data, and a post-hoc test was used to compare the means. The data was displayed as mean ± SD. The probability of chance (P-value) was used to assess the significance of the data; a value of P < 0.001 was interpreted as highly significant, a value of P < 0.05 was interpreted as significant, and a value of P > 0.05 was interpreted as non-significant.

### Statistical analysis

For each group, the results except for morphometric studies were expressed as mean ± SD. GraphPad Prism, software application version 5.0 (2007), Inc., CA, USA, was used for statistical analysis. When comparing more than two groups, Tukey’s multiple comparison test was performed after the one-way ANOVA was used to determine the statistical difference between the groups. Statistical significance was defined as P values of 0.05.

## Results

There were no significant differences seen between the vehicle-treated group and the control group across all evaluated parameters. Thus, for comparisons with the model-RF and RF-ZA groups, the control group was used. There was no mortality in all groups throughout the 12 week study.

### Effects on kidney index

According to the data presented in Table 1, the kidney index in the model-RF group showed a substantial rise of 59.7% compared to the control group, with a statistically significant difference (P < 0.0001). It is noteworthy that the administration of ZA at a dosage of 50 µg/kg/week resulted in a substantial (P < 0.0001) reduction in the kidney index by 32.3%.

**Table 1:** Effect of ZA (50 µg/kg/week) on MBP, kidney index, serum creatinine, BUN, uACR in a CCl_4_ induced RF rat model.

	Control	Model-RF	RF-ZA
MBP (mmHg)	66 ± 2.1	24 ± 0.97***	63 ± 2.3^###^
Kidney index	0.62 ± 0.005	0.99 ± 0.072***	0.67 ± 0.039^###^
uACR	11 ± 0.39	37 ± 1.2***	14 ± 1.7^###^
Serum creatinine (nmol/ml)	2.1 ± 0.14	7.6 ± 0.90 ***	2.5 ± 0.72^###^
BUN (nmol/ml)	231 ± 4.6	676 ± 40***	251 ± 18^###^

Data are mean ± S.D. n = 10 rats per group.

*ZA* zoledronic acid, *RF* renal fibrosis, *MBP* mean blood pressure, *uACR* urinary albumin to creatinine ratio, *BUN* blood urea nitrogen.

***P < 0.0001 significant compared to control group.

^###^P < 0.0001 significant compared to model-RF group by one-way ANOVA with Tukey’s multiple comparison test.

### Effects on renal functions

#### Effects on Mean blood pressure (MBP)

The model-RF group had a significant reduction (P < 0.0001) in MBP compared to the control group, with a decrease of 63.7%. The administration of ZA at a dosage of 50 µg/kg/week effectively (P < 0.0001) prevented a reduction in the mean arterial pressure (MBP) by 162.5% (Table 1).

#### Urinary albumin to creatinine ratio (uACR)

The level of uACR was found to be considerably higher (P < 0.0001) in the model-RF group compared to the control group, with an increase of 236.4%. The group treated with RF-ZA exhibited a statistically significant (P < 0.0001) decrease in uACR compared to the model-RF group, with a reduction of 62.2% (Table 1).

#### Serum creatinine and blood urea nitrogen (BUN) levels

The model-RF group exhibited a statistically significant (P < 0.0001) elevation in serum creatinine and BUN levels compared to the control group, with increases of 261.9% and 192.6% respectively. Conversely, the RF-ZA treated group demonstrated a statistically significant (P < 0.0001) reduction in serum creatinine and BUN levels compared to the model group, with decreases of 67.1% and 62.9% respectively (Table 1).

### Effects on oxidative stress markers: malondialdehyde (MDA) and catalase levels

According to the data shown in Table 2, the model-RF group exhibited a statistically significant increase (P < 0.0001) in the concentration of MDA in comparison to the control group, with a rise of 354.5%. The group treated with RF-ZA had a statistically significant (P < 0.0001) decrease in MDA levels compared to the model-RF group, with a reduction of 72.7%. In contrast, there was a substantial drop (P < 0.0001) in the catalase level observed in the model-RF group compared to the control group, with a decrease of 68.9%. Additionally, the administration of ZA to rats resulted in a large rise (P < 0.0001) in the renal content of catalase compared to the model-RF group, with an increase of 190.5%.

**Table 2:** Effect of ZA (50 µg/kg/week) on MDA, catalase, TNF-α, TGF-β, FPP and expression of NF-kB and FPPS in kidney tissues of a CCl_4_ induced RF rat model.

	Control	Model- RF	RF-ZA
MDA (nmol/mg)	0.33 ± 0.03	1.5 ± 0.06***	0.41 ± 0.09^###^
Catalase (mU/mg)	3.016 ± 0.204	0.938 ± 0.119***	2.725 ± 0.275^###^
TNF-α (pg/mg)	29.2 ± 3.3	184 ± 24.8***	47.9 ± 4.57^###^
TGF-β (pg/mg)	57.4 ± 3.2	336 ± 34.2***	61.6 ± 16.4^###^
FPP (ng/mg)	215 ± 24	391 ± 16***	235 ± 32^###^
FPPS	1.0 ± 0.01	5.6 ± 1.20***	1.3 ± 0.27^###^
NF-kB	1.1 ± 0.1	8.7 ± 2.1***	1.2 ± 0.1^###^

Data are mean ± S.D. n = 10 rats per group.

*ZA* zoledronic acid, *RF* renal fibrosis, *MDA* malondialdehyde, *TNF-α* tumor necrosis factor-alpha, *TGF-β* transforming growth factor-β, *FPP* farnesyl pyrophosphate, *NFκB* nuclear factor-κB, *FPPS* farnesyl pyrophosphate synthase.

***P < 0.0001 significant compared to control group.

^###^P < 0.0001 significant compared to model-RF group by one-way ANOVA with Tukey’s multiple comparison test.

### Effects on tumor necrosis factor-α (TNF-α) and transforming growth factor-β (TGF-β) levels

In the model-RF group, there was a substantial increase (P < 0.0001) in the levels of renal TNF-α and TGF-β compared to the control group, with an increase of 530.1% and 485.4% respectively. The administration of ZA to rats resulted in a significant reduction (P < 0.0001) in the renal levels of TNF-α and TGF-β, with a decrease of 74.0% and 81.7% respectively, as compared to the model-RF group (Table 2).

### Effects on renal farnesyl pyrophosphate (FPP) protein level

According to the data shown in Table 2, there was a substantial increase (P < 0.0001) in the renal FPP level seen in the model-RF group compared to the control group, with an increase of 81.9%. The group treated with RF-ZA had a statistically significant (P < 0.0001) reduction in renal FPP level by 39.9% compared to the model-RF group.

### Effects on renal farnesyl pyrophosphate synthetase (FPPS) and nuclear factor-kappa B (NF-kB) gene expressions

It was found that renal FPPS and NF-kB expression were significantly (P < 0.0001) upregulated in the model-RF group compared to the control group by 460% and 690.9%, respectively. RF-ZA treated group showed a significant (P < 0.0001) down-regulation of renal FPPS and NF-kB expression in comparison to the model-RF group by 76.8% and 86.2% respectively (Table 2).

### Histological and histomorphometric results

Examination of the control and vehicle groups showed similar findings and represented as “control group”. Statistical results revealed non-significant differences between the control group and the vehicle group (control groups) in all measured parameters.

**Figure 2:**
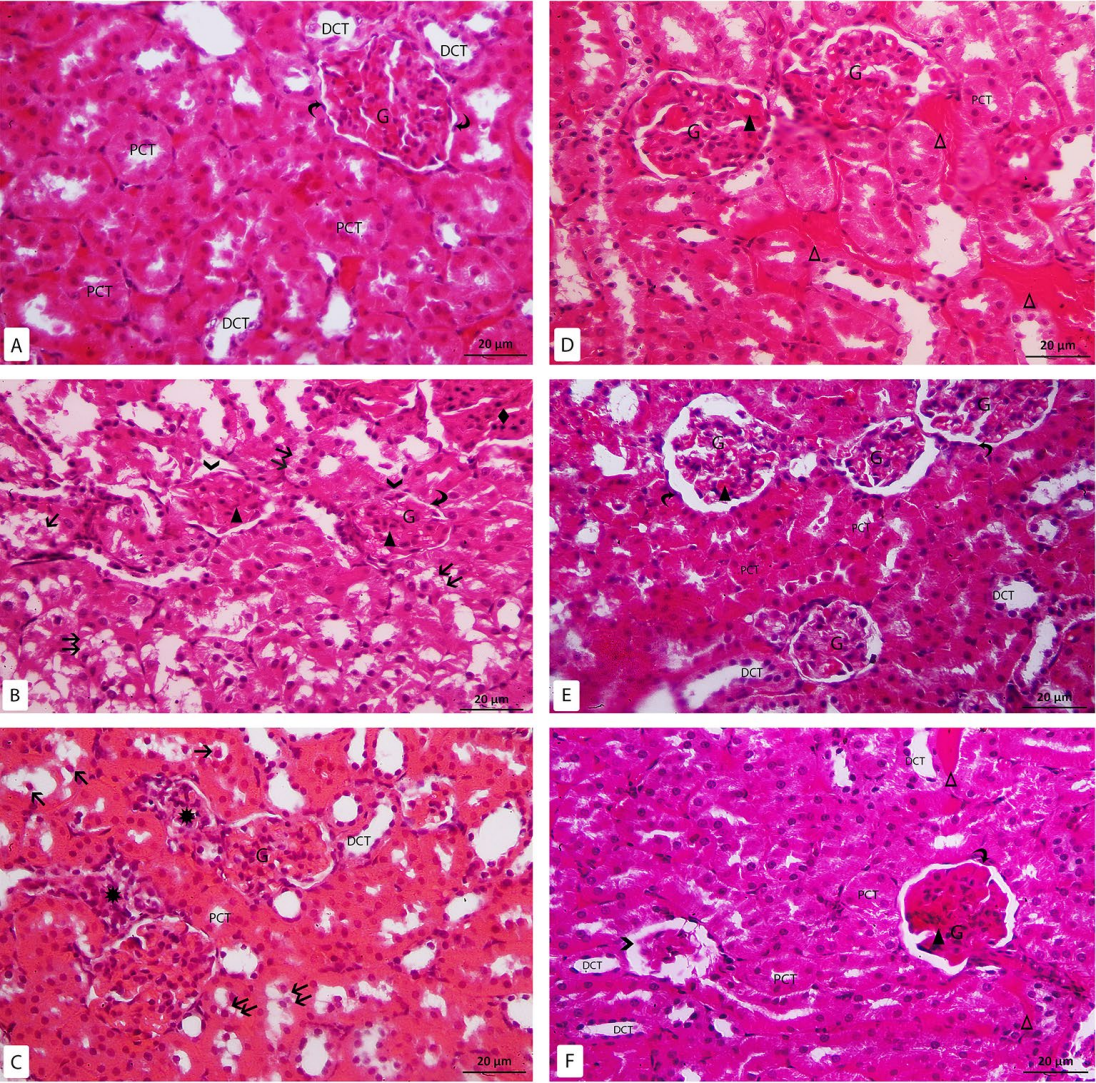
H&E-stained sections X400: (**A**) control group: the glomerulus (G), parietal layer of Bowman’s capsule (curved arrow), proximal convoluted tubules (PCT), distal convoluted tubules (DCT). (**B**–**D**) Model-RF group: the glomerulus (G), parietal layer of bowman’s capsule (curved arrow), disrupted parietal layer of Bowman’s capsule ( >), proximal convoluted tubules (PCT), distal convoluted tubules (DCT), sloughed tubular cells (↑), vacuolated tubular cells (↑↑), mononuclear cell infiltration (*), congested glomerular (▲) and peritubular capillaries (Δ), acidophilic area with pyknotic nuclei (♦). (**E**,**F**) RF-ZA group: the glomerulus (G), parietal layer of bowman’s capsule (curved arrow), proximal convoluted tubules (PCT), distal convoluted tubules (DCT), shrunken glomerulus ( >), congested glomerular (▲) and peritubular capillaries (Δ).

H & E-stained slides of the control group showed that the renal cortex was consisted of the renal corpuscles surrounded with closely packed renal tubules. The renal corpuscles were seen with tuft of capillaries surrounded by Bowman’s capsule. Bowman’s capsule was composed of visceral and parietal layers with Bowman’s space in-between. The parietal layer was consisted of simple squamous cells. The proximal convoluted tubules (PCTs) were composed of cuboidal cells with spherical basal nuclei and heavy acidophilic cytoplasm. While the distal convoluted tubules (DCTs) were formed of low cuboidal cells with pale eosinophilic cytoplasm and round central to apical nuclei. The DCTs had wider lumina than the PCTs (Fig. 2A). In Model group, the histopathological changes were observed in the cortex including the glomeruli and the tubules. Most renal corpuscles were seen with irregular outline. Some corpuscles were seen with disrupted parietal layer of Bowman’s capsule. Most renal tubules were dilated with sloughing of their epithelial cells inside the lumina with intraluminal acidophilic material. Vacuolated renal tubular cells were also noticed. Some tubules also showed pyknotic nuclei. Mononuclear cell infiltration was frequently seen in the renal interstitium. Dilated and congested glomerular and peritubular capillaries were also noticed (Fig. 2B–D). In zoledronate group, some areas showed apparent typical renal corpuscles surrounded with closely packed renal tubules. Other areas showed shrunken glomeruli. Some vacuolated tubular cells were also noticed. Glomerular and peritubular vascular congestion was occasionally seen (Fig. 2E, F).

**Figure 3:**
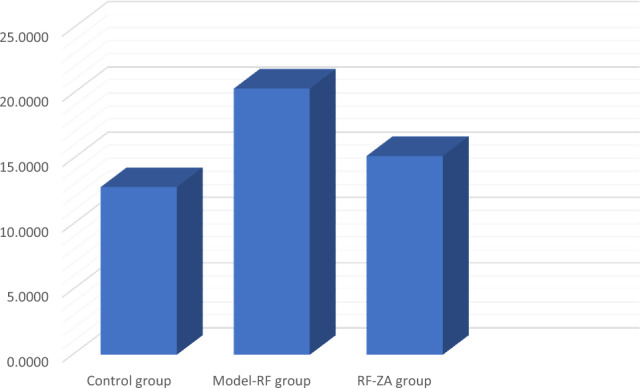
Histogram showing the PCT diameter (µm) in different groups.

The mean tubular diameter of the PCTs showed a highly significant difference between the different groups (f = 22.931, P < 0.001) by using one-way ANOVA. Compared to the control group, Post Hoc LSD showed a highly significant increase in the mean tubular diameter in the model group (P < 0.001). Also, there was significant increase in the mean tubular diameter in the RF-ZA group (P = 0.048). Meanwhile, there was a highly significant decrease in the mean tubular diameter in the RF-ZA group compared to the model-RF group (P < 0.001) (Table 3 and Fig. 3).

**Table 3:** Effect of ZA (50 µg/kg/week) on area % of collagen fibers, area % of α SMA, area % of caspase 3 and, area % of IL-6 expression and PCT diameter and their relation in a CCI_4_ induced RF rat model.

Variable	Control group	Model-RF	RF-ZA group
PCT diameter (µm)	12.87 ± 2.86	20.41 ± 3.79***	15.25 ± 1.81^###^
Area % of collagen fibers	1.69 ± 0.22	7.06 ± 1.22***	2.66 ± 0.61^###^
Area % of caspase 3	0.37 ± 0 .12	2.32 ± 0 .71***	0.94 ± 0. 19^###^
Area % of IL-6	0.37 ± 0 .11	4.79 ± 1.50***	1.52 ± 0.48^###^
Area % of α SMA	0.19 ± 0.06	4.67 ± 1.48***	1.70 ± 0.39^###^

Data are mean ± S.D. n = 10 rats per group.

*ZA* zoledronic acid, *RF* renal fibrosis, *α-SMA* α-smooth muscle actin, *IL-6* interleukin-6, *PCT* proximal convoluted tubule.

***P < 0.0001 significant compared to control group.

^###^P < 0.0001 significant compared to model-RF group by one-way ANOVA with Tukey’s test.

**Figure 4:**
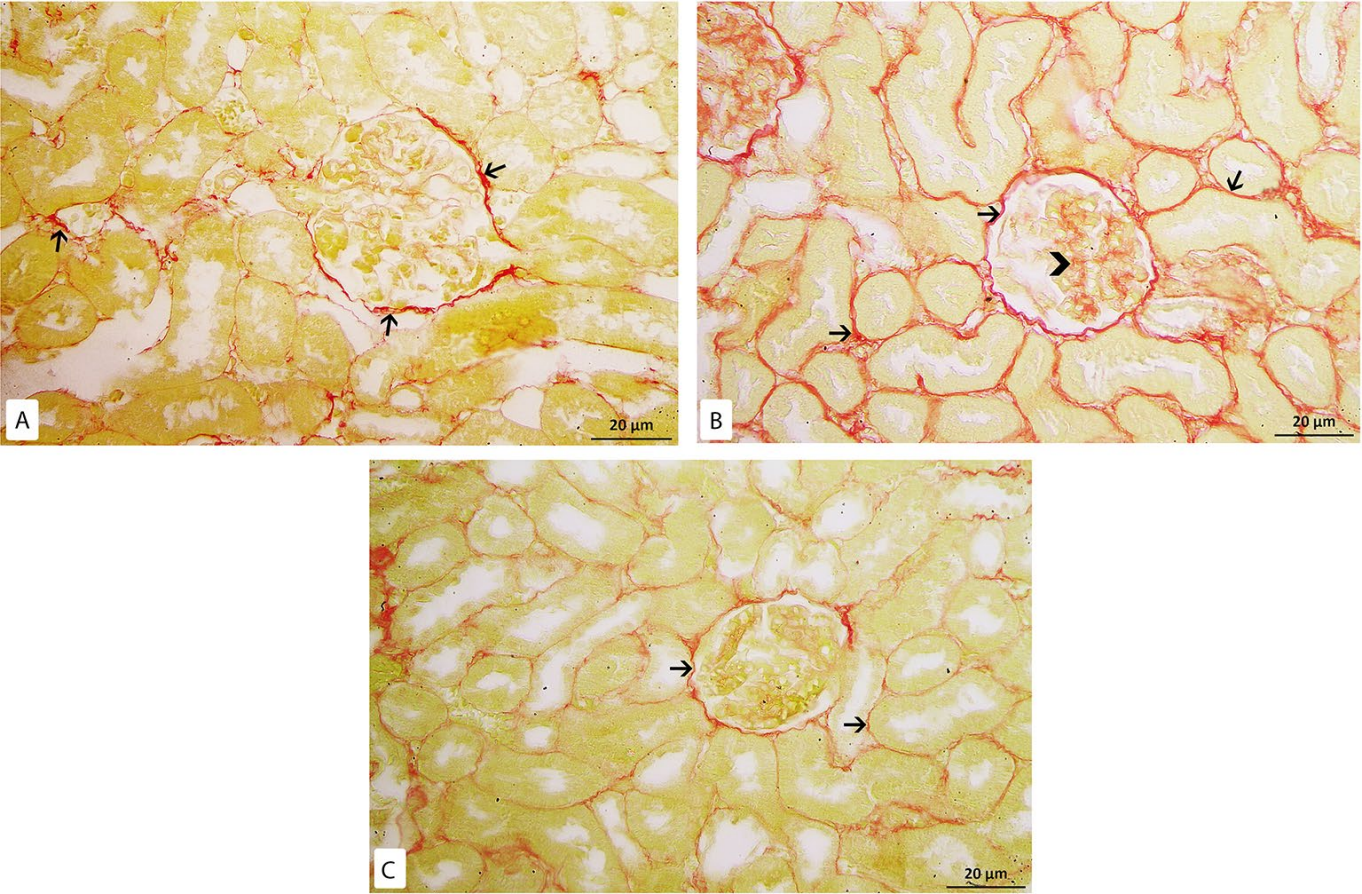
Picrosirius red X400. (**A**) Control group: few collagen fibers (↑) around renal corpuscle and renal tubules. (**B**) Model-RF group: massive collagen fibers (↑) around renal corpuscles and renal tubules. Increased amount of collagen fibers is also seen inside renal glomeruli ( >) (**C**) RF-ZA group: mild collagen fibers (↑) around renal corpuscle and renal tubules. No collagen fibers inside renal glomeruli.

**Figure 5:**
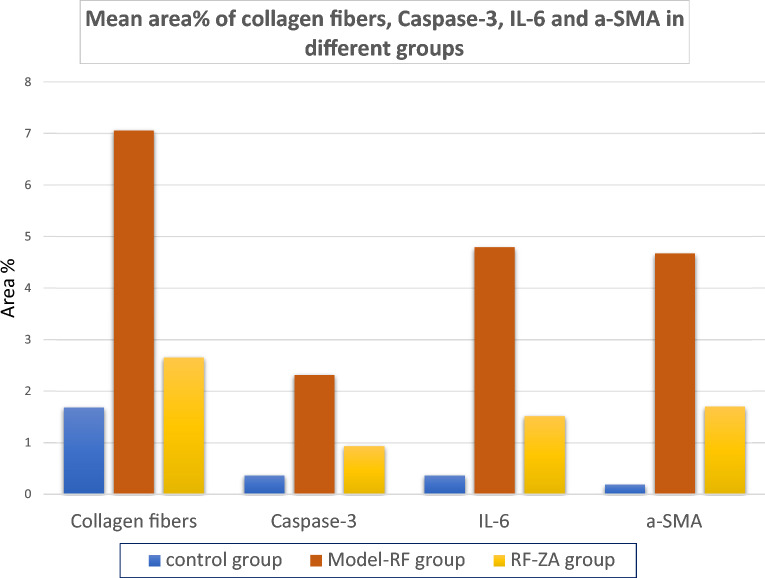
Histogram showing the mean area % of collagen fibers, caspase-3, IL-6 and α-SMA in different groups.

Kidney sections were stained with picrosirius red stain for demonstration of collagen fibers. By examination of the control group slides, few collagen fibers were revealed around renal corpuscles, renal tubules and around blood vessels (Fig. 4A). While in the model-RF, increased collagen fibers were noticed around blood vessels, renal tubules, and renal corpuscles. The amount of the collagen fibers was also observed to be increased inside most renal glomeruli (Fig. 4B) with a highly significant increase in the mean area % of collagen fibers compared to the control group (P < 0.001). In RF-ZA group, decreased collagen fibers were noticed with highly significant decrease in the mean area % of collagen fibers compared to model-RF group (P < 0.001) (Fig. 4C). A highly significant difference in the mean area % of collagen fibers was noticed between the different groups (f = 131.500, P < 0.001). Post Hoc LSD showed There was also a significant increase in the mean area % of collagen fibers in the RF-ZA group compared to the control group (P = 0.004) (Table 3 and Fig. 5).

**Figure 6:**
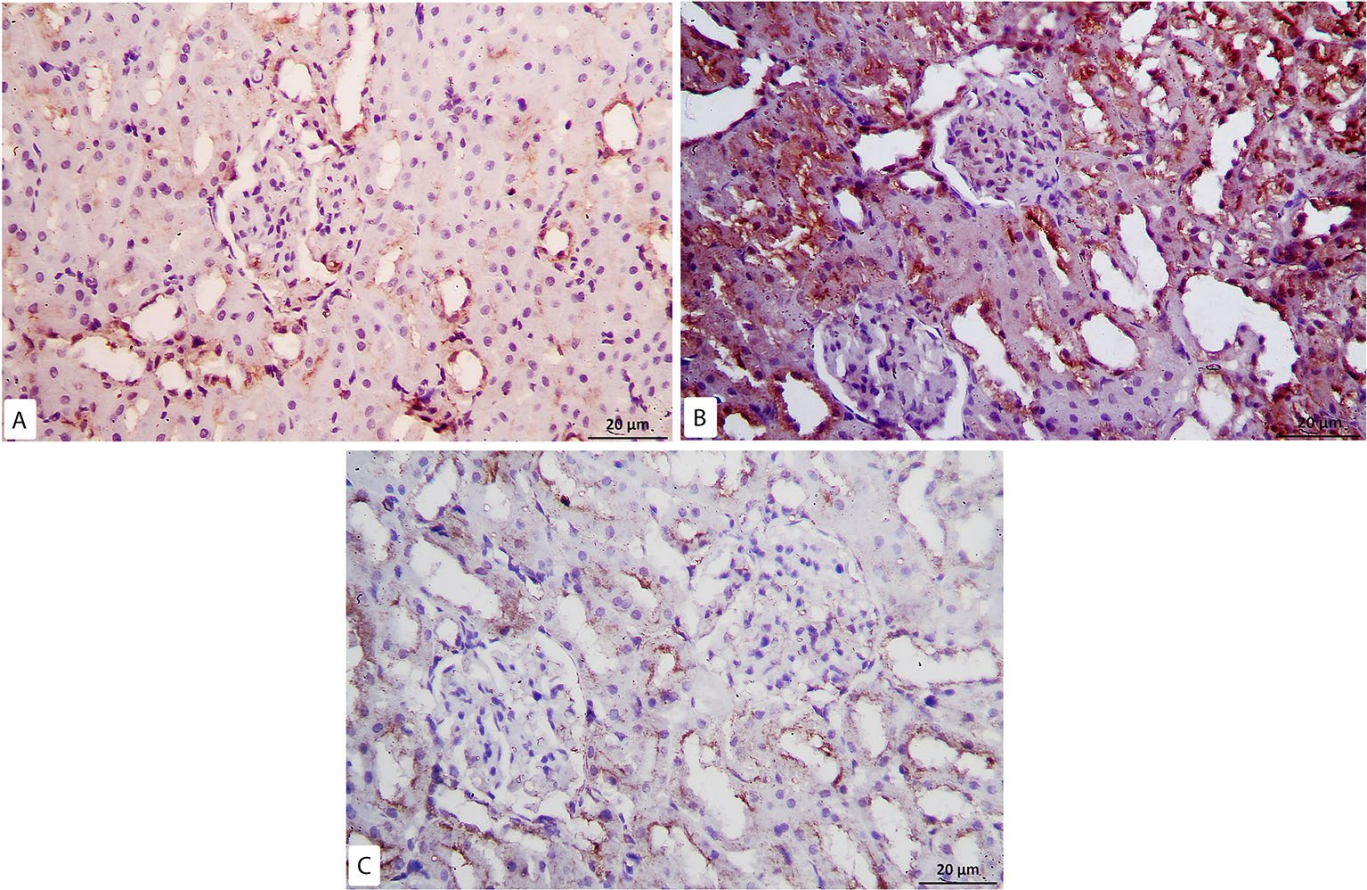
Caspase 3 Immunohistochemistry X400. (**A**) Control group: minimal reaction to caspase 3 in some tubular cells. (**B**) Model-RF Group: intense reaction to caspase 3 in most renal tubules. [(**C**) RF-ZA group: mild reaction to caspase 3 in some renal tubules.

Examination of immunohistochemical caspase-3 slides of control group revealed minimal positive reaction in the cytoplasm of some tubular cells and cells inside few renal corpuscles (Fig. 6A), whereas, in model-RF group, intense positive cytoplasmic immune reaction was seen in most renal tubules with a highly significant increase in the mean area % of caspase-3 compared to the control (P < 0.001) (Fig. 6B). While RF-ZA group showed mild positive cytoplasmic caspase-3 reaction in some renal tubules with highly significant decrease in the mean area % of caspase-3 compared to the Model-RF (P < 0.001) (Fig. 6C). A highly significant difference in the mean area % of caspase-3 was noticed between the different groups (f = 56.597, P < 0.001). Post Hoc LSD also showed a significant increase in the mean area % of caspase-3 in the RF-ZA group compared to the control group (P = 0.002). (Table 3 and Fig. 5).

**Figure 7:**
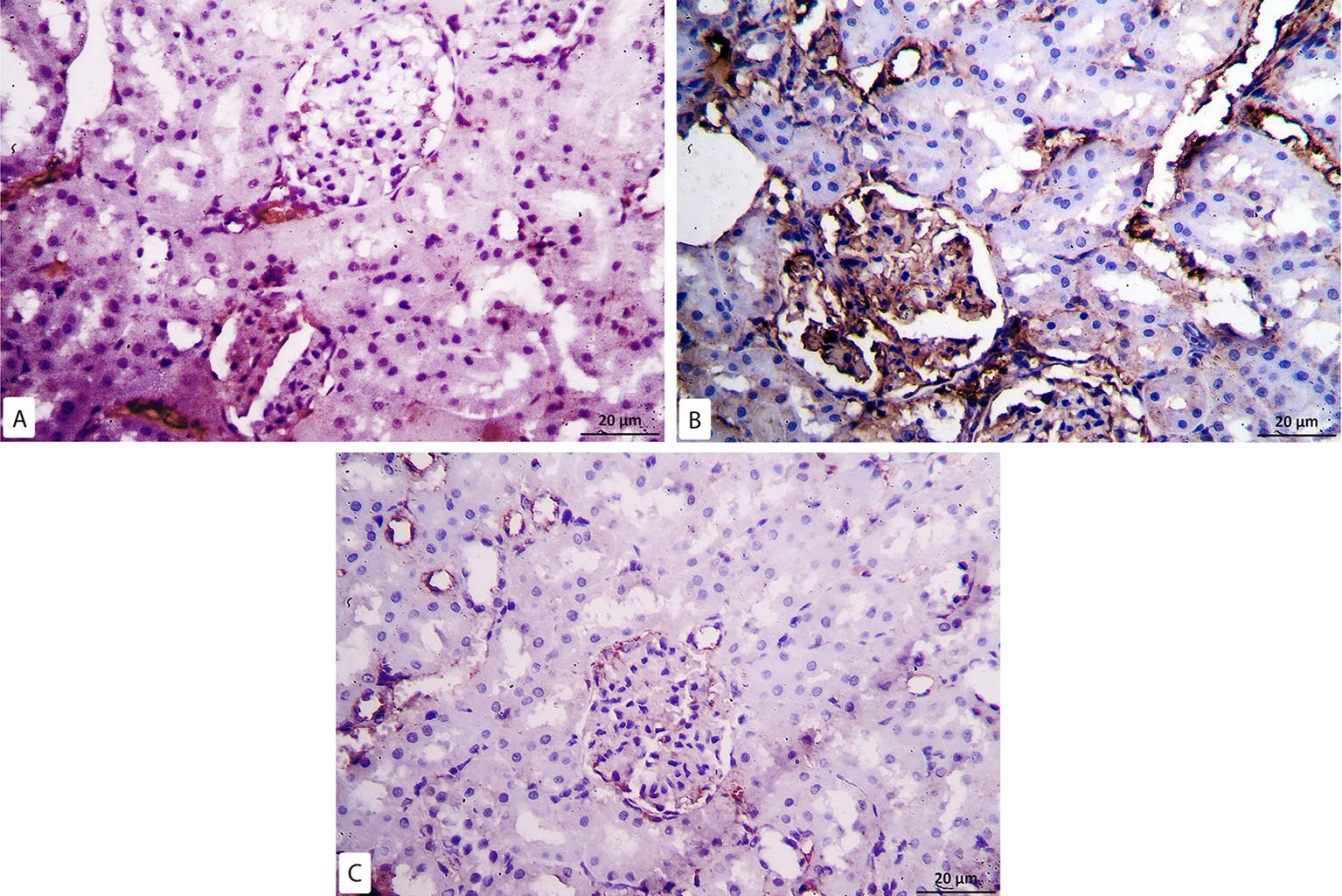
IL6 Immunohistochemistry X400. (**A**) Control group: minimal IL6 expression. (**B**) Model-RF Group: intense IL6 expression. (**C**) RF-ZA group: mild IL6 expression.

Regarding IL-6 immunohistochemical reaction, minimal positive reaction was noticed in the control group which was confined mostly to the peritubular capillaries (Fig. 7A). While the model group sections revealed intense positive expression with a highly significant increase in the mean area % of IL-6 compared to the control rats (P < 0.001). The reaction was noticed in the cytoplasm of cells inside the glomeruli and in the renal interstitium mostly in peritubular capillaries (Fig. 7B). In RF-ZA group, mild reaction was noticed in some cells inside renal glomeruli and surrounding renal tubules with a highly significant decrease in the mean area % of IL-6 compared to the model-RF group (P < 0.001) (Fig. 7C). A highly significant difference in the mean area % of IL-6 was noticed between the different groups (f = 66.877, P < 0.001). There was also significant increase in the mean area % of IL-6 in the RF-ZA group compared to the control group (P = 0.002) (Table 3 and Fig. 5).

**Figure 8:**
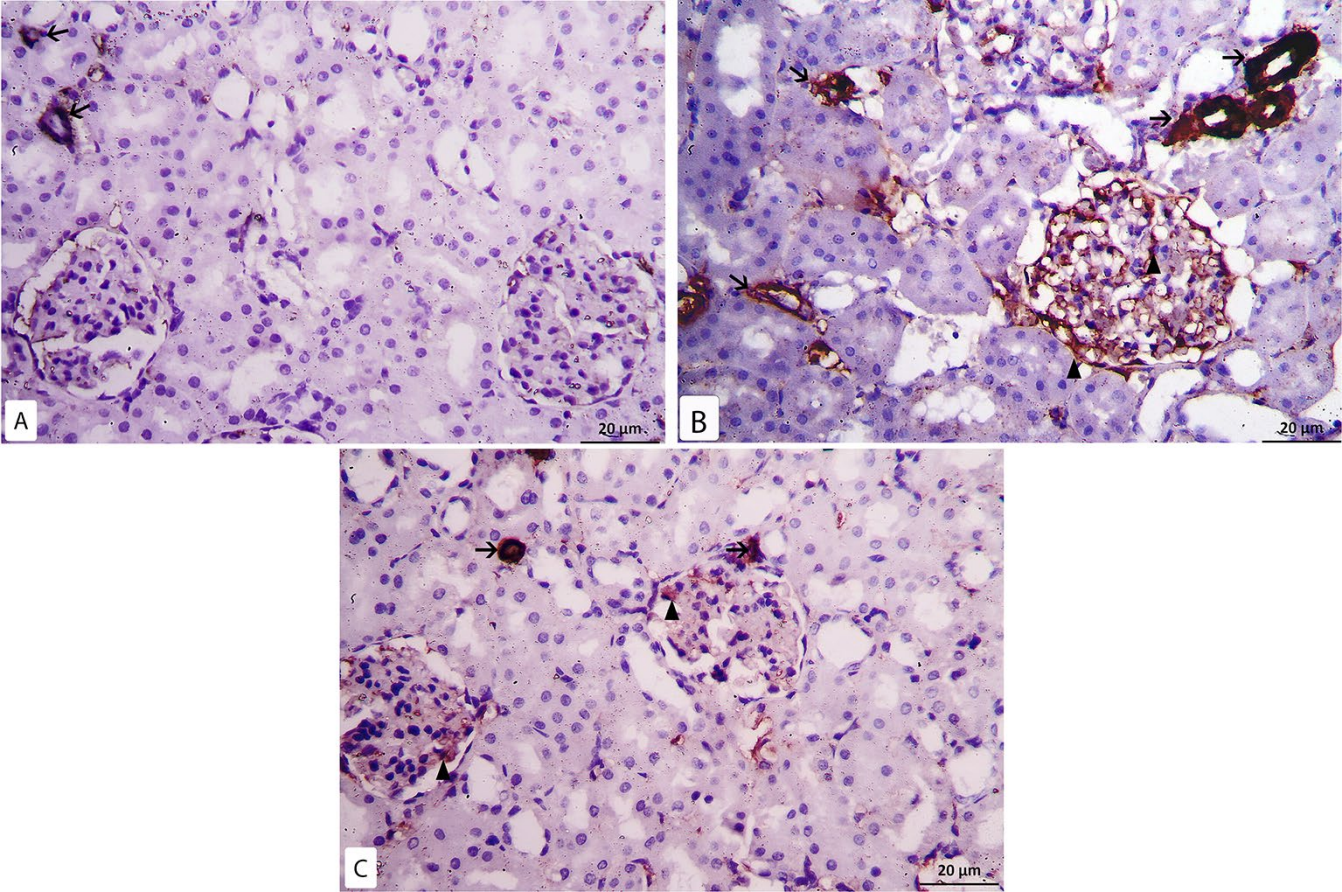
α-SMA Immunohistochemistry X400. (**A**) Control group: minimal reaction to α-SMA in the wall of blood vessels (↑), negative reaction is seen in the renal glomeruli. (**B**) Model-RF group: intense reaction to α- SMA in the wall of blood vessels (↑) and inside the glomerulus (▲). (**C**) RF-ZA group: mild reaction to α-SMA in the wall of blood vessels (↑) and inside the glomerulus (▲).

In α- SMA immunohistochemical sections of the control group minimal positive reaction was detected in the walls of the blood vessels. While the glomeruli and the tubules revealed negative reaction (Fig. 8A). In model-RF group intense positive reaction was noticed with a high significant increase in the mean area % of α-SMA compared to the control group (P < 0.001). The reaction was seen inside most renal glomeruli, in the walls of blood vessels and in some renal tubular epithelial cells (Fig. 8B). In RF-ZA group, mild positive reaction was seen with high significant decrease in the mean area % of α-SMA compared to model-RF group (P < 0.001). The reaction was seen in the walls of the blood vessels and inside some renal glomeruli, while negative reaction was seen in renal tubules (Fig. 8C). A highly significant difference in the mean area % of α-SMA was noticed between the different groups (f = 74.471, P < 0.001). There was also high significant increase in the mean area % of α-SMA in RF-ZA group compared to the control group (P < 0.001) (Table 3 and Fig. 5).

## Discussion

In this study, we induced renal fibrosis (RF) in rats by using carbon tetrachloride (CCl_4_). Research showed that CCl_4_ causes oxidative stress and triggers the production of cytokines such as tumor necrosis factor-alpha (TNFα) and growth factors like transforming growth factor-β (TGF-β) in renal tissues. CCl_4_ enhances apoptosis by stimulating the caspase pathway, resulting in renal damage^21^. Additionally, CCl_4_ boosts protein prenylation, such as protein farnesylation and geranylgeranylation, by upregulating the production of farnesyl pyrophosphate (FPP) and geranylgeranyl pyrophosphate (GGPP) through the stimulation of reactive oxygen species (ROS)^7,25^.

The study indicated that zoledronic acid (ZA) at 50 µg/kg/week has a protective effect on the kidneys in a rat model of renal failure produced by CCl_4_. ZA markedly enhanced kidney functioning. It reduced elevated levels of urine albumin–creatinine ratio (uACR), serum creatinine, and blood urea nitrogen (BUN). The results included a notable reduction in the protein levels of tumor necrosis factor-alpha (TNF-α), transforming growth factor-β (TGF-β), and malondialdehyde (MDA), along with a considerable increase in catalase protein level in renal tissues. The beneficial benefits may be attributed to ZA’s capacity to hinder the renal production of farnesyl pyrophosphate synthase (FPPS) and farnesyl pyrophosphate (FPP), leading to improved protein prenylation and decreased expression of nuclear factor-κB (NF-κB).

Protein prenylation is involved in cardiovascular, bone, and neurodegenerative illnesses. It is crucial in the advancement and growth of fibrosis by activating the NF κB signaling pathway. Hence, prenylation has emerged as an appealing target for inhibitors with potential therapeutic benefits^4,26^.

FPPS is a vital enzyme in the mevalonate pathway responsible for synthesizing isoprenoid intermediates like FPP^27^. FPP is necessary for the prenylation of RhoGTPases that trigger the NF κB signaling pathway, which is activated in fibrotic diseases^4^. Previous studies have confirmed that increased FPPS expression leads to the development of liver and lung fibrosis as well as heart hypertrophy^28–31^.

Renal fibrosis, a critical pathological alteration in the progression of chronic kidney disease to end-stage renal disease, is heavily influenced by the NF-κB signaling system. The primary function of NF-κB is to augment the secretion of TNF-κ, IL-6, and TGF-β, resulting in notable fibrotic buildup and infiltration of inflammatory cells into the renal tissue. It was reported that macrophages infilteration and polarization aggravate renal inflammation and fibrosis^32,33^. Suppressing macrophages polarization and fibroblasts activation attenuates extracellular matrix protein production, and preserves kidney function^34,35^. In rheumatoid factor, IL-6 plays a pivotal role. The expression of the IL-6 gene is regulated by the NF-κB pathway. Tumor necrosis factor-alpha (TNF-α), predominantly found in renal tubular cells, can trigger an inflammatory response and lead to renal fibrosis ^12,22,36^.

Increasing amounts of reactive oxygen species (ROS) and isoprenoid substrates stimulate renal fibrosis via boosting TGF-β signaling via the NF-κB signaling pathway^37,38^. TGF-β stimulates the attraction, specialization, and persistence of myofibroblasts identified by the presence of α-smooth muscle actin (α-SMA) and increased matrix accumulation, resulting in renal fibrosis. Activating transforming growth factor-beta (TGF-β) is essential for inducing epithelial-to-mesenchymal transition (EMT) in renal tubular cells, hence leading to the progression of renal interstitial fibrosis^39,40^. Therefore, targeting of NF-κB through inhibition of protein prenylation by the inhibition of FPPS has entered the spotlight.

It was reported that ZA, by inhibiting FPPS, reduced NF-κB signaling, decreased inflammatory cell recruitment, inhibited myofibroblast transition, reduced migration of pro-fibrotic cell populations, and eventually ameliorated lung fibrosis^13^, hepatic fibrosis^14^ and myocardial fibrosis^41^. Göbel et al.^42^, did a study that provides evidence for the efficacy of ZA treatment in suppressing the activity of TGF-β, TNF-α, and IL-6 in ovarian cancer cells through the inhibition of the FPPS.

The study found that continuous exposure to CCl_4_ caused structural changes in kidney tissues and led to a notable rise in the average area of caspase-3 in renal tubules and α-smooth muscle actin (α-SMA) in the peritubular capillaries and renal glomeruli. Additionally, there was a notable rise in the average area % of collagen fiber within renal glomeruli and in the renal interstitium. The results were consistent with prior experimental investigations that found CCl_4_ treatment caused kidney damage, cell death, and increased production of the apoptosis-related protein caspase-3^22,43^. Previous investigations have shown that in certain kidney diseases, mesangial cells undergo a transformation into myofibroblasts. Alpha-smooth muscle actin (α-SMA) is a biomarker for myofibroblasts, which are cells that overproduce collagen^44,45^.

ZA at a modest dose of 50 µg/kg/week improved the histological changes in renal tissues caused by CCl_4_. ZA suppressed the expression of interleukin-6 (IL-6), α-SMA, and caspase-3 via reducing FPPS expression and decreasing FPP protein levels in renal tissues. The results of our study provide evidence for the efficacy of statins in inhibiting the production of isoprenoids, specifically FPP and GGPP, showing encouraging anti-fibrotic effects^46,47^. Statin disrupts isoprenoid synthesis, which inhibits NF-κB activation and TNF-α generation, leading to contrasting inflammatory and fibrosis effects^48^.

Furthermore, this study found that model-RF rats exhibited a low mean blood pressure (MBP). ZA (50 µg/kg/week) markedly enhanced MBP in ZA-RF rats compared to model-RF rats. The increase in MBP may be because to the improvement of renal functioning by ZA. The kidneys regulate blood pressure through mechanisms such as fluid balance, electrolyte management, and the renin–angiotensin–aldosterone system. End-stage renal disease can lead to reduced generation and effectiveness of angiotensin II, causing vasodilation and lowering blood pressure^49^.

Conflicting research results exist regarding the effect of ZA on renal function. Zoledronic acid (ZA) is the most effective bisphosphonate for treating bone problems related to cancer. ZA is commonly used when patients have impaired kidney function due to diseases including hypertension, diabetes, age-related renal insufficiency, or cancer -induce renal disease. Moreover, it is characterized by widespread approval, enabling safe and lasting integration^16,50^. Previous clinical trials found no link between yearly infusions of ZA and decline in kidney function in postmenopausal patients with osteoporosis^51,52^. Other studies suggest that ZA may lead to nephrotoxicity. In one of these trials, a higher dose of 3 mg/kg was provided compared to the level utilized in our investigation^53^. A different study found that ZA treatment resulted in decreased kidney function in rats with renal ischemia–reperfusion injury, but not in normal rats like in our investigation^15^. Bergner and his colleagues detected reduced renal function in rats with ZA in a dose of 1 mg/kg for a longer duration (27 weeks) than the duration of our study^54^. In patients treated with ZA at recommended doses and infusion rates, renal toxicity is rare, as confirmed by long-term data from clinical trials^55–58^.

The study used a dose of 50 µg/kg/week of ZA for 12 weeks to replicate the clinical dose given to human patients with osteoporosis. Additionally, it was shown that a ZA dose exceeding 200 µg/kg/week is deemed high for Wistar rats. The 4 mg ZA medication dosage given monthly to cancer patients is appropriate for preventing metastases^59–61^. The ZA dose (< 200 µg/kg/week) is considered minimal and is comparable to the doses administered intravenously for treating osteoporosis patients^62,63^. The nephrotoxicity of ZA depends on the dosage and duration of treatment. It is common in patients with severe renal impairment. Zoledronic acid is contraindicated only in cases of substantial renal insufficiency (stage IV–V, GFR 30 ml/min)^16^. The incidence of hypertension, diabetes, obesity, and other conditions leading to kidney damage and CKD is increasing annually, making CKD a major health concern^12^. Therefore, there is a requirement for pharmacological therapy that is both efficient and secure. ZA may be a viable treatment for preventing the progression of chronic kidney disease (CKD) and renal failure (RF) in high-risk patients.

Although our study has important implications, it also has limits that must be acknowledged. First, we focused on the effect of ZA on FPPS, FPP and NFκB parameters in protein prenylation pathway. However, they are not the only possible mediators of protein prenylation pathway. Therefore, the effect of ZA on other parameters that have an important role in protein prenylation induced fibrosis should be evaluated as RhoGTPases which are involved in activating NFκB signaling pathway. Determination of kidney injury score is one of important parameters that should be evaluated. Second, the effect of this dose of ZA should be evaluated in rats with normal renal function. The unavailability of some resources due to financial constricts were important obstacles in our study.

## Conclusion

Our findings demonstrate that ZA administration at dose of (50 µg kg/week) showed efficacy against CCl_4_-induced RF through prevention of protein prenylation mainly by decreasing the expression of FPPS, FPP, and NF-κB. Consequently, there was a decrease in FPP, NF-Κb, oxidative stress (MDA), inflammation (TNF-α, and IL-6), apoptosis (caspase-3) and fibrosis (TGF-β and α-SMA) induced by CCl_4_ in renal tissues. These data support the use of ZA in clinical trials to prevent RF.

### Supplementary Information

Supplementary Figures.

## Data Availability

Data is available from the corresponding author on reasonable request.

## Abbreviations

BUN: Blood urea nitrogen
CCl_4_: Carbon tetrachloride
CKD: Chronic kidney disease
CT: Critical threshold
Ct: Cycle threshold
EMT: Epithelial-to-mesenchymal transition
FPP: Farnesyl pyrophosphate
FPPS: Farnesyl pyrophosphate synthase
H&E: Hematoxylin and eosin
i.p.: Intraperitoneal
IL-6: Interleukin-6
MBP: Mean arterial blood pressure
MDA: Malondialdehyde
NF-Κb: Nuclear factor-Kb
PBS: Phosphate buffer saline
PCT: Proximal convoluted tubule
RF: Renal fibrosis
ROS: Reactive oxygen species
RQ: Relative quantitation
RT-PCR: Real time PCR
RT-qPCR: Real-time quantitative polymerase chain reaction
TGF-β: Transforming growth factor-β
TNF-α: Tumor necrosis factor-alpha
uACR: Urine albumin–creatinine ratio
ZA: Zoledronic acid
α-SMA: α-Smooth muscle actin

## References

[1] Huang, R., Fu, P. & Ma, L. Kidney fibrosis: From mechanisms to therapeutic medicines. *Sig. Transduct. Target Ther.* **8**, 129. 10.1038/s41392-023-01379-7 (2023). 10.1038/s41392-023-01379-7 PMC10023808 36932062

[2] Wang, C., Li, S. W., Zhong, X., Liu, B. C. & Lv, L. L. An update on renal fibrosis: From mechanisms to therapeutic strategies with a focus on extracellular vesicles. *Kidney Res. Clin. Pract.* **42**(2), 174–187. 10.23876/j.krcp.22.159 (2023). 37037480 10.23876/j.krcp.22.159 PMC10085720

[3] Vaidya, S. R. & Aeddula, N. R. Chronic Kidney Disease. [Updated 2022 Oct 24]. In *StatPearls* [Internet]. Available from: https://www.ncbi.nlm.nih.gov/books/NBK535404/ (StatPearls Publishing, Treasure Island (FL), 2024).

[4] Ung, C. Y., Onoufriadis, A., Parsons, M., McGrath, J. & Shaw, T. Metabolic perturbations in fibrosis disease. *Int. J. Biochem. Cell Biol.*10.1016/j.biocel.2021.106073 (2021). 34461262 10.1016/j.biocel.2021.106073

[5] Manaswiyoungkul, P., de Araujo, E. D. & Gunning, P. T. Targeting prenylation inhibition through the mevalonate pathway. *RSC Med. Chem.* **11**(1), 51–71. 10.1039/c9md00442d (2019). 33479604 10.1039/c9md00442d PMC7485146

[6] Dhar, M. K., Koul, A. & Kaul, S. Farnesyl pyrophosphate synthase: A key enzyme in isoprenoid biosynthetic pathway and potential molecular target for drug development. *New Biotechnol.* **30**, 114–123. 10.1016/j.nbt.2012.07.001 (2013). 10.1016/j.nbt.2012.07.001 22842101

[7] Jiang, D., Chen, Y., Zhu, Y., Fu, G. & Xu, S. Expression of key enzymes in the mevalonate pathway are altered in monocrotaline-induced pulmonary arterial hypertension in rats. *Mol. Med. Rep.* **16**(6), 9593–9600. 10.3892/mmr.2017.7798 (2017). 29039598 10.3892/mmr.2017.7798

[8] Hooff, G. P., Wood, W. G., Müller, W. E. & Eckert, G. P. Isoprenoids, small GTPases and Alzheimer’s disease. *Biochim. Biophys. Acta* **1801**(8), 896–905. 10.1016/j.bbalip.2010.03.014 (2010). 20382260 10.1016/j.bbalip.2010.03.014 PMC2886181

[9] Tong, L. & Tergaonkar, V. Rho protein GTPases and their interactions with NFκB: Crossroads of inflammation and matrix biology. *Biosci. Rep.* **34**(3), e00115. 10.1042/BSR20140021 (2014). 24877606 10.1042/BSR20140021 PMC4069681

[10] Liu, T. *et al.* NF-κB signaling in inflammation. *Sig. Transduct. Target Ther.* **2**, 17023. 10.1038/sigtrans.2017.23 (2017). 10.1038/sigtrans.2017.23 PMC5661633 29158945

[11] White, S., Lin, L. & Hu, K. NF-κB and tPA signaling in kidney and other diseases. *Cells* **9**(6), 1348. 10.3390/cells9061348 (2020). 32485860 10.3390/cells9061348 PMC7348801

[12] Wang, Y. *et al.* Mechanism of dioscin ameliorating renal fibrosis through NF-κB signaling pathway-mediated inflammatory response. *Mol. Med. Rep.* **27**(4), 93. 10.3892/mmr.2023.12980 (2023). 36960871 10.3892/mmr.2023.12980 PMC10073814

[13] Tanner, L. *et al.* Zoledronic acid targeting of the mevalonate pathway causes reduced cell recruitment and attenuates pulmonary fibrosis. *Front. Pharmacol.*10.3389/fphar.2022.899469 (2022). 35721132 10.3389/fphar.2022.899469 PMC9201219

[14] Mohamed, R. H., Tarek, M., Hamam, G. G. & Ezzat, S. F. Zoledronic acid prevents the hepatic changes associated with high fat diet in rats; the potential role of mevalonic acid pathway in nonalcoholic steatohepatitis. *Eur. J. Pharmacol.*10.1016/j.ejphar.2019.172469 (2019). 31233751 10.1016/j.ejphar.2019.172469

[15] Sert, İU. *et al.* The role of vitamin E in the prevention of zoledronic acid-induced nephrotoxicity in rats: A light and electron microscopy study. *Arch. Med. Sci.* **14**(2), 381–387 (2018). 29593813 10.5114/aoms.2016.60227 PMC5868662

[16] Fixen, C. W. & Fixen, D. R. Renal safety of zoledronic acid for osteoporosis in adults 75 years and older. *Osteoporos. Int.* **33**(11), 2417–2422. 10.1007/s00198-022-06499-4 (2022). 35829757 10.1007/s00198-022-06499-4

[17] Percie du Sert, N. *et al.* The ARRIVE guidelines 2.0: Updated guidelines for reporting animal research. *Br. J. Pharmacol.* **177**(16), 3617–3624. 10.1111/bph.15193 (2020). 32662519 10.1111/bph.15193 PMC7393194

[18] Novartis Pharmaceuticals Australia Pty Ltd. Aclasta product information. (2009).

[19] Reagan-Shaw, S., Nihal, M. & Ahmad, N. Dose translation from animal to human studies. *FASEB J.* **22**(3), 659–661 (2008). 17942826 10.1096/fj.07-9574LSF

[20] Sengupta, P. The laboratory rat: Relating its age with human’s. *Int. J. Prev. Med.* **4**(6), 624–630 (2013). 23930179 PMC3733029

[21] Doi, K., Kurabe, S., Shimazu, N. & Inagaki, M. Systemic histopathology of rats with CCl_4_-induced hepatic cirrhosis. *Lab. Anim.* **25**(1), 21–25 (1991). 2010972 10.1258/002367791780808121

[22] Jie-Qiong, Ma., Zhang, Y.-J., Tian, Z.-K. & Liu, C.-M. Bixin attenuates carbon tetrachloride induced oxidative stress, inflammation and fibrosis in kidney by regulating the Nrf2/TLR4/MyD88 and PPAR-γ/TGF-β1/Smad3 pathway. *Int. Immunopharmacol.* **90**, 107117. 10.1016/j.intimp.2020.107117 (2021). 33162346 10.1016/j.intimp.2020.107117

[23] Livak, K. J. & Schmittgen, T. D. Analysis of relative gene expression data using real-time quantitative PCR and the 2−ΔΔCT method. *Methods* **25**(4), 402–408. 10.1006/meth.2001.1262 (2001). 11846609 10.1006/meth.2001.1262

[24] Survarna, K. S., Layton, C. & Bancroft, J. D. *Bancroft’s Theory and Practice of Histological Techniques* 8th edn, 126–138 (Churchill Livingstone, Elsevier, 2013).

[25] Chen, W. B. *et al.* GGPPS deficiency aggravates CCl_4_-induced liver injury by inducing hepatocyte apoptosis. *FEBS Lett.* **589**(10), 1119–1126. 10.1016/j.febslet.2015.03.015 (2015). 25819439 10.1016/j.febslet.2015.03.015

[26] Jeong, A., Suazo, K. F., Wood, W. G., Distefano, M. D. & Li, L. Isoprenoids and protein prenylation: Implications in the pathogenesis and therapeutic intervention of Alzheimer’s disease. *Crit. Rev. Biochem. Mol. Biol.* **53**(3), 279–310. 10.1080/10409238.2018.1458070 (2018). 29718780 10.1080/10409238.2018.1458070 PMC6101676

[27] Mullen, P. J., Yu, R., Longo, J., Archer, M. C. & Penn, L. Z. The interplay between cell signalling and the mevalonate pathway in cancer. *Nat. Rev. Cancer* **16**(11), 718–731 (2016). 27562463 10.1038/nrc.2016.76

[28] Yang, J. *et al.* Cardiac-specific overexpression of farnesyl pyrophosphate synthase induces cardiac hypertrophy and dysfunction in mice. *Cardiovasc. Res.* **97**, 490–499. 10.1093/cvr/cvs347 (2013). 23180723 10.1093/cvr/cvs347

[29] Larson-Casey, J. L., Murthy, S., Ryan, A. J. & Carter, A. B. Modulation of the mevalonate pathway by Akt regulates macrophage survival and development of pulmonary fibrosis. *J. Biol. Chem.* **289**(52), 36204–36219 (2014). 25378391 10.1074/jbc.M114.593285 PMC4276883

[30] Zhao, C. Z. *et al.* Inhibition of farnesyl pyrophosphate synthase improves pressure overload induced chronic cardiac remodeling. *Sci. Rep.* **6**, 39186. 10.1038/srep39186 (2016). 28008986 10.1038/srep39186 PMC5180215

[31] Zhao, Y., Wu, T. Y., Zhao, M. F. & Li, C. J. The balance of protein farnesylation and geranylgeranylation during the progression of nonalcoholic fatty liver disease. *J. Biol. Chem.* **295**(15), 5152–5162. 10.1074/jbc.REV119.008897 (2020). 32139507 10.1074/jbc.REV119.008897 PMC7152775

[32] Jiao, B. *et al.* STAT6 deficiency attenuates myeloid fibroblast activation and macrophage polarization in experimental folic acid nephropathy. *Cells* **10**, 3057. 10.3390/cells10113057 (2021). 34831280 10.3390/cells10113057 PMC8623460

[33] An, C. *et al.* Myeloid PTEN deficiency aggravates renal inflammation and fibrosis in angiotensin II-induced hypertension. *J. Cell. Physiol.* **237**(1), 983–991. 10.1002/jcp.30574 (2022). 34515350 10.1002/jcp.30574 PMC8810675

[34] Jiao, B. *et al.* Pharmacological inhibition of STAT6 ameliorates myeloid fibroblast activation and alternative macrophage polarization in renal fibrosis. *Front. Immunol.* **26**(12), 735014. 10.3389/fimmu.2021.735014 (2021). 10.3389/fimmu.2021.735014 PMC8426438 34512669

[35] An, C. *et al.* Jumonji domain-containing protein-3 (JMJD3) promotes myeloid fibroblast activation and macrophage polarization in kidney fibrosis. *Br. J. Pharmacol.* **180**(17), 2250–2265. 10.1111/bph.16096 (2023). 37076137 10.1111/bph.16096 PMC12641593

[36] Sanz, A. B. *et al.* TWEAK activates the non-canonical NFkappaB pathway in murine renal tubular cells: Modulation of CCL21. *PLoS One* **5**(1), e8955. 10.1371/journal.pone.0008955 (2010). 20126461 10.1371/journal.pone.0008955 PMC2813291

[37] Patel, S. *et al.* Inhibitory effect of statins on renal epithelial-to-mesenchymal transition. *Am. J. Nephrol.* **26**(4), 381–387. 10.1159/000094780 (2006). 16873994 10.1159/000094780

[38] Ma, Z. *et al.* Lovastatin alleviates endothelial-to-mesenchymal transition in glomeruli via suppression of oxidative stress and TGF-β1 signaling. *Front. Pharmacol.* **8**, 473 (2017). 28769803 10.3389/fphar.2017.00473 PMC5513942

[39] Sureshbabu, A., Muhsin, S. A. & Choi, M. E. TGF-β signaling in the kidney: Profibrotic and protective effects. *Am. J. Physiol. Renal Physiol.* **310**(7), F596–F606. 10.1152/ajprenal.00365.2015 (2016). 26739888 10.1152/ajprenal.00365.2015 PMC4824143

[40] Zhou, T. *et al.* Runt-related transcription factor 1 (RUNX1) promotes TGF-β-induced renal tubular epithelial-to-mesenchymal transition (EMT) and renal fibrosis through the PI3K subunit p110δ. *EBioMedicine* **31**, 217–225 (2018). 29759484 10.1016/j.ebiom.2018.04.023 PMC6013935

[41] Xu, H. *et al.* Inhibition of the mevalonate pathway improves myocardial fibrosis. *Exp. Ther. Med.* **3**, 224. 10.3892/etm.2021.9655 (2021). 10.3892/etm.2021.9655 PMC7851600 33603833

[42] Göbel, A. *et al.* Anti-tumor effects of mevalonate pathway inhibition in ovarian cancer. *BMC Cancer* **20**, 1–17 (2020). 10.1186/s12885-020-07164-x PMC7388525 32727400

[43] Ma, J. Q., Liu, C. M. & Yang, W. Protective effect of rutin against carbon tetrachloride-induced oxidative stress, inflammation and apoptosis in mouse kidney associated with the ceramide, MAPKs, p53 and calpain activities. *Chem. Biol. Interact.* **286**, 26–33. 10.1016/j.cbi.2018.03.003 (2018). 29522708 10.1016/j.cbi.2018.03.003

[44] Fu, R. G. *et al.* Inhibition of the K+ channel K(Ca)3.1 reduces TGF-β1-induced premature senescence, myofibroblast phenotype transition and proliferation of mesangial cells. *PLoS One* **9**, e87410 (2014). 24489911 10.1371/journal.pone.0087410 PMC3905019

[45] Oujo, B. *et al.* L-endoglin overexpression increases renal fibrosis after unilateral ureteral obstruction. *PLoS One* **9**, e110365 (2014). 25313562 10.1371/journal.pone.0110365 PMC4196986

[46] Santos, D. M. *et al.* Screening for YAP inhibitors identifies statins as modulators of fibrosis. *Am. J. Respir. Cell Mol. Biol.* **62**(4), 479–492 (2020). 31944822 10.1165/rcmb.2019-0296OC PMC7110981

[47] Leiya, K., Pei, K., Guangwei, L. & Shuang, W. Progress of statin therapy in the treatment of idiopathic pulmonary fibrosis. *Oxidative Med. Cell. Longev.*10.1155/2022/6197219 (2022). 10.1155/2022/6197219 PMC8957418 35345828

[48] Sheridan, A., Wheeler-Jones, C. P. D. & Gage, M. C. The immunomodulatory effects of statins on macrophages. *Immuno* **2**(2), 317–343. 10.3390/immuno2020021 (2022). 10.3390/immuno2020021

[49] Wadei, H. M. & Textor, S. C. The role of the kidney in regulating arterial blood pressure. *Nat. Rev. Nephrol.* **8**(10), 602–609 (2012). 22926246 10.1038/nrneph.2012.191

[50] Isla, D., Afonso, R., Bosch-Barrera, J. & Martínez, N. Zoledronic acid in lung cancer with bone metastases: A review. *Expert Rev. Anticancer Ther.* **13**(4), 421–426. 10.1586/era.13.15 (2013). 23560837 10.1586/era.13.15

[51] Black, D. M. *et al.* Once-yearly zoledronic acid for treatment of postmenopausal osteoporosis. *N. Engl. J. Med.* **356**(18), 1809–1822. 10.1056/NEJMoa067312 (2007). 17476007 10.1056/NEJMoa067312

[52] Boonen, S. *et al.* Renal safety of annual zoledronic acid infusions in osteoporotic postmenopausal women. *Kidney Int.* **74**(5), 641–648. 10.1038/ki.2008.193 (2008). 18509324 10.1038/ki.2008.193

[53] Bouomrani, S., Regaïeg, N., Nefoussi, M. & Trabelsi, S. Zoledronic acid-induced acute renal failure in multiple myeloma. *JOJ Case Stud.* **9**(2), 555757. 10.19080/JOJCS.2018.09.555757 (2018). 10.19080/JOJCS.2018.09.555757

[54] Bergner, R., Siegrist, B., Gretz, N., Pohlmeyer-Esch, G. & Kränzlin, B. Nephrotoxicity of ibandronate and zoledronate in Wistar rats with normal renal function and after unilateral nephrectomy. *Pharmacol. Res.*10.1016/j.phrs.2015.04.016 (2015). 25976681 10.1016/j.phrs.2015.04.016

[55] JT, C. Renal failure with the use of zoledronic acid. *N. Engl. J. Med.* **349**, 1676–1679 (2004). 10.1056/NEJM200310233491721 14573746

[56] Munier, A. *et al.* Zoledronic acid and renal toxicity: Data from French adverse effect reporting database. *Ann. Pharmacother.* **39**(7–8), 1194–1197 (2005). 15956222 10.1345/aph.1E589

[57] Perazella, M. A. & Markowitz, G. S. Bisphosphonate nephrotoxicity. *Kidney Int.* **74**(11), 1385–1393 (2008). 18685574 10.1038/ki.2008.356

[58] Pozzi, S. & Raje, N. The role of bisphosphonates in multiple myeloma: Mechanisms, side effects, and the future. *Oncol.* **16**(5), 651–662 (2011). 10.1634/theoncologist.2010-0225 PMC3228190 21493759

[59] Pozzi, S. *et al.* High-dose zoledronic acid impacts bone remodeling with effects on osteoblastic lineage and bone mechanical properties. *Clin. Cancer Res.* **15**(18), 5829–5839. 10.1158/1078-0432.CCR-09-0426 (2009). 19737962 10.1158/1078-0432.CCR-09-0426

[60] de Sousa, F. R. N. *et al.* The effect of high concentration of zoledronic acid on tooth induced movement and its repercussion on root, periodontal ligament and alveolar bone tissues in rats. *Sci. Rep.* **11**(1), 7672. 10.1038/s41598-021-87375-9 (2021). 33828221 10.1038/s41598-021-87375-9 PMC8027035

[61] Soares, M. Q. S. *et al.* High doses of zoledronic acid induce differential effects on femur and jawbone microstructure. *Clin. Exp. Dent. Res.* **8**(6), 1487–1495. 10.1002/cre2.643 (2022). 35933703 10.1002/cre2.643 PMC9760133

[62] Barros Silva, P. G. *et al.* Effect of different doses of zoledronic acid in establishing of bisphosphonate-related osteonecrosis. *Arch. Oral Biol.*10.1016/j.archoralbio.2015.05.015 (2015). 10.1016/j.archoralbio.2015.05.015 26093347

[63] Shaheen, M. Y. *et al.* Effect of systemic zoledronic acid dosing regimens on bone regeneration in osteoporotic rats. *Appl. Sci.* **11**(4), 1906. 10.3390/app11041906 (2021). 10.3390/app11041906

