# Supplementary figures and images for: The role of protein prenylation inhibition through targeting FPPS by zoledronic acid in the prevention of renal fibrosis in rats
Source: Sci Rep. 2024 Aug 7;14:18283. doi: 10.1038/s41598-024-68303-z (PMC11306734; doi:10.1038/s41598-024-68303-z)

**Supplementary figures**
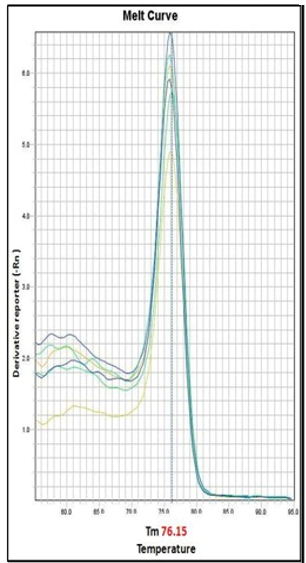


**Figure S1: Melting curve of FPPS gene**


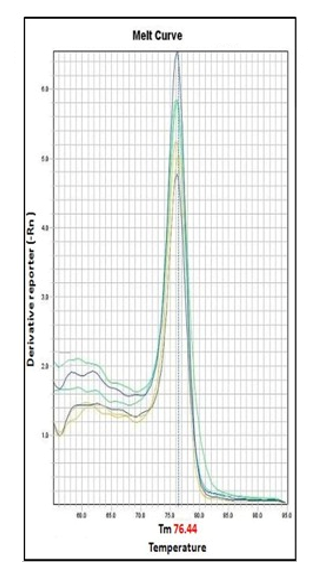


**Figure S2: Melting curve of NF-κB gene**

Supplement: Supplementary file 1 — Supplementary Figures. [file 41598_2024_68303_MOESM1_ESM.docx]
